# Implementation of the participatory approach for supervisors to prevent sick leave: a process evaluation

**DOI:** 10.1007/s00420-016-1118-6

**Published:** 2016-03-12

**Authors:** R. A. Kraaijeveld, F. G. Schaafsma, S. M. Ketelaar, C. R. L. Boot, U. Bültmann, J. R. Anema

**Affiliations:** Department of Public and Occupational Health, EMGO+ Institute for Health and Care Research, VU University Medical Center, Amsterdam, The Netherlands; Research Center for Insurance Medicine, Collaboration Between AMC-UMCG-UWV-VUmc, Amsterdam, The Netherlands; Body@Work, Research Center Physical Activity, Work and Health, TNO-VU University Medical Center, Amsterdam, The Netherlands; Department of Health Sciences, Community and Occupational Medicine, University Medical Center Groningen, University of Groningen, Groningen, The Netherlands

**Keywords:** Participatory approach, Prevention, Process evaluation, Sick leave, Supervisors, Workplace

## Abstract

**Purpose:**

To perform a process evaluation of a multifaceted strategy to implement the participatory approach for supervisors to prevent sick leave in three organisations.

**Methods:**

The implementation strategy incorporated a working group meeting with stakeholder representatives, supervisor training, and optional supervisor coaching. Context, recruitment, reach, dose delivered, dose received, fidelity, and satisfaction with the strategy were assessed at organisational and supervisor level using questionnaires and registration forms.

**Results:**

At least 4 out of 6 stakeholders were represented in the working group meetings, and 11 % (*n* = 116) of supervisors could be reached. The working group meetings and supervisor training were delivered and received as planned and were well appreciated within all three organisations. Three supervisors made use of coaching. At 6-month follow-up, 11 out of 41 supervisors (27 %) indicated that they had applied the participatory approach at least one time.

**Conclusion:**

The implementation strategy was largely carried out as intended. However, reach of both supervisors and department managers should be improved. Future studies should consider targeting employees with the strategy.

## Introduction

The participatory approach (PA) originates from participatory ergonomics developed to implement ergonomic measures from the bottom up in a workplace (Rivilis et al. 2008; Driessen et al. 2010). Over the years, this stepwise approach has also been expanded towards a workplace intervention protocol for supervisors and employees to identify and solve barriers to return to work (RTW). This protocol is generally applied by an occupational health professional (OHP) such as a trained occupational health nurse. This approach has been shown to be effective to shorten sick-leave episodes of employees with health complaints (Loisel et al. 1997; Anema et al. 2007; Lambeek et al. 2010; van Oostrom et al. 2010). For example, Lambeek et al. (2010) demonstrated that the PA within an integrated care approach for sick-listed employees due to low back pain decreased the time to sustainable RTW by 120 days. The PA might also be useful for the prevention of sick leave. In most organisations in the Netherlands, supervisors play a key role in the management, but also prevention of sick leave (Schreuder et al. 2013). Therefore, to target prevention of sick leave, application of the PA by supervisors could be valuable. To our knowledge, however, the PA is not generally applied by supervisors in Dutch organisations. In 2012, we set up a study to implement the application of the PA by supervisors within three different organisations (Kraaijeveld et al. 2013). For this study, the PA was shaped into a workplace intervention protocol for supervisors and employees to jointly identify and solve work functioning problems due to health complaints to prevent sick leave.

When implementing the PA within an organisation, aiming to prevent sick leave, barriers were expected at different organisational levels. Earlier research has shown that a multifaceted strategy targeting different organisational levels is necessary to implement interventions within organisations (Grol and Grimshaw 2003; Rasmussen et al. 2013). Therefore, a multifaceted implementation strategy was developed that aimed to target different organisational levels and incorporated several elements: (1) a working group meeting with stakeholder representatives from the organisation, (2) supervisor training by OHPs regarding the application of the PA, and (3) the supervisor coaching by an OHP (optional). By using this multifaceted strategy, we aimed to tackle barriers to implementation of the PA at both the organisational and the supervisor level (Kraaijeveld et al. 2013). Possible barriers at organisational level are organisational policies and attitudes towards employees at risk of sick leave (van Oostrom et al. 2009). For example, human resources (HR) may not facilitate modified work for employees at risk of sick leave. The working group in our implementation strategy was used to tailor the PA protocol to the organisation and to tackle organisational barriers for supervisors to apply the PA. At supervisor level, possible barriers are a lack of communication skills and the belief that employees with health complaints only need rest to recover (van Oostrom et al. 2009; Shaw et al. 2006). The training and coaching in our implementation strategy were aimed towards enhancing supervisors’ communication skills and encouraging supervisors to apply the PA if an employee is at risk of sick leave.

Conducting a process evaluation can help to determine the extent to which an implementation strategy is carried out as intended, as well as to examine whether the strategy is feasible in daily practice and whether it can be carried out on a large scale (Grol and Grimshaw 2003; Murta et al. 2007). Moreover, process data may also give insight into how and why the multifaceted strategy of the PA is effective (or not), for whom and in which context (Durlak and DuPre 2008; Linnan and Steckler 2002; Nielsen 2013). Thus, a process evaluation offers information to improve the implementation strategy of a workplace intervention.

This paper describes the process evaluation of the multifaceted implementation strategy of the PA by using the Linnan and Steckler’s framework (2002). This framework has been applied in comparable studies (Arends et al. 2014; Driessen et al. 2010; van der Meer et al. 2014), and has proved to be a useful guide to conduct process evaluations of implementation studies (Murta et al. 2007). The aim of this process evaluation was threefold: (1) to examine whether the multifaceted implementation strategy of the PA was carried out as intended, (2) to identify barriers to the application of the PA by supervisors, and (3) to examine the extent to which the PA was applied by supervisors.

## Methods

### Study design

The process evaluation was performed alongside a cluster-randomised controlled trial (RCT) (Kraaijeveld et al. 2013). Participating supervisors were randomly allocated at department level to either the intervention group or the control group. Departments in the intervention group received the multifaceted implementation strategy of the PA, while departments in the control group received only a minimal implementation strategy consisting of distribution of written information on PA. This process evaluation focuses on the 19 departments in the intervention group and examines the period between baseline and 6-month follow-up. The Medical Ethics Committee of the VU University Medical Center Amsterdam assessed the RCT study protocol and judged that written informed consent of participants was not required.

### Study population

Three organisations participated in the study: a steel factory, a university medical centre, and a university. Based on earlier studies on implementation of the PA, the following six stakeholders were selected to take part in the working groups: supervisors, employees, managers at department level, HR professionals, occupational health professionals (OHPs), and occupational physicians (OPs) (Driessen et al. 2010; van Oostrom et al. 2007). Occupational health professionals in this study were occupational health nurses, company labour experts, or company social workers. Stakeholder representatives for the working groups and supervisors for the supervisor training were recruited within the three organisations. Only stakeholder representatives who participated in the working group meetings and supervisors of the intervention group who received the training were included in the process evaluation.

### Study context

The Dutch Gatekeeper Improvement Act states that the employer and the employee are primarily responsible for RTW of the employee. Employers appoint a case manager for sick-leave management, who is responsible for coordination of the RTW process and (administrative) actions that are required by law. In most organisations in the Netherlands, the direct supervisor is responsible for case management.

### Multifaceted implementation strategy

The multifaceted implementation strategy was carried out after the baseline measurement and incorporated three components, described below.

#### Working group meeting

Representatives of the six selected stakeholders were invited to participate in a 2-h working group meeting chaired by an in-company OHP. During this meeting the following topics were discussed: (1) information about how to identify risk of sick leave, (2) when and how supervisors should discuss the risk of sick leave with the employee, (3) content and application of the workplace intervention protocol of the PA, and (4) possible organisational barriers to supervisors’ application of the PA and how to deal with these barriers. The information given by stakeholder representatives was used to customise the workplace intervention protocol and supervisor training for each organisation.

#### Supervisor training

Supervisors were invited to participate in a 4-h training from in-company OHPs during working time. The in-company OHPs were previously trained by the researchers (RAK, FGS, JRA) on how to train the supervisors. In each training session, a maximum of eight supervisors participated. During the training the following topics were discussed: (1) information about how to identify risk of sick leave, (2) when and how supervisors should discuss risk of sick leave with the employee, (3) content of workplace intervention protocol of the PA, and (4) application of the workplace intervention protocol of the PA. The training included an oral presentation, group discussions about the main topics, and role-playing to practise the application of the workplace intervention protocol. The protocol instructs to hold three supervisor–employee meetings in which the supervisor acts as a process leader. In the first meeting, the supervisor addresses the employee’s risk of sick leave and proposes to use the PA. In preparation of the second meeting, both the supervisor and the employee separately make an inventory of the employee’s work tasks and activities, prioritise the employee’s work functioning problems, and think of possible solutions for the two most important work functioning problems. In the second meeting, the supervisor and employee discuss the two most important work functioning problems together with the possible solutions and assess the applicability of these solutions. They agree on an action plan to realise these solutions; if they cannot reach agreement, an in-company OHP can be asked to act as process leader. Subsequently, solutions are prepared and realised. In the third meeting, the supervisor and employee evaluate the action plan and the realised solutions. After the training, supervisors could enrol in a follow-up training if necessary.

Although the workplace intervention protocol is primarily targeted at preventing sick leave, supervisors were also instructed to apply the protocol to sick-listed employees (i.e. to jointly identify and solve barriers to RTW).

#### Supervisor coaching

After the training, supervisors had the option to ask an in-company OHP as a coach to prepare or guide the application of the PA at any time during the follow-up period. For example, if supervisors found it too difficult to act both as the supervisor and as a process leader, they could ask an in-company OHP to act as process leader.

### Process variables

Using the framework of Linnan and Steckler (2002), seven process variables were assessed: context, recruitment, reach, dose delivered, dose received, fidelity, and satisfaction. These process variables were assessed at organisational level (working group meeting) and supervisor level (training and coaching). In addition, the extent to which the PA was applied and barriers to application of the PA were assessed.

#### Context

Context refers to organisational factors that could directly or indirectly affect the implementation of workplace interventions (Murta et al. 2007). The following factors of the participating organisations were taken into account: company size, sick-leave policies, sick-leave rates, and the type of in-company OHPs that delivered the supervisor training and coaching.

#### Recruitment

##### Organisational level

Recruitment was defined as the procedures that were used to recruit stakeholder representatives for the study.

##### Supervisor level

At the supervisor level, recruitment was defined as the procedures that were used to recruit supervisors for the study. Based on our sample size calculation, we targeted to recruit at least 107 supervisors equally divided over the 3 organisations (Kraaijeveld et al. 2013).

#### Reach

##### Organisational level

Reach was defined as the percentage of approached stakeholder representatives that attended the working group meetings. In addition, as all six stakeholders were meant to be represented at each working group meeting, the proportion of stakeholders that were represented per organisation was calculated. Furthermore, reasons for non-participation were registered.

##### Supervisor level

Reach was defined as the percentage of approached supervisors that enrolled in the study, i.e. supervisors who filled out the baseline questionnaire. In addition, the number of supervisors that were allocated to the intervention group was registered, as well as the number of supervisors that attended the training and the number of supervisors that were coached during the follow-up. Reasons for non-participation were also registered.

#### Dose delivered

##### Organisational level

Dose delivered was defined as the number of working group meetings that were actually delivered: within each organisation one 2-h working group meeting had to be delivered by an in-company OHP.

##### Supervisor level

The number of supervisor training sessions and coaching sessions that were delivered by the in-company OHPs was used as parameter for dose delivered at the supervisor level: within each organisation the 4-h training was required and the 2-h follow-up training and coaching were optional.

#### Dose received

##### Organisational level

Dose received was defined as the extent to which stakeholder representatives reported to have participated actively during the working group meeting. Participation was measured with the following item: “During the working group meeting I had a meaningful contribution”. Directly after the working group meeting, stakeholder representatives were asked to rate this item on an evaluation form. Response categories ranged from 1 (totally disagree) to 5 (totally agree). The percentage of stakeholder representatives that agreed or totally agreed with this statement was calculated.

##### Supervisor level

The extent to which supervisors reported that the training had contributed to their understanding how and when to use the PA was used as parameter for dose delivered at the supervisor level. This was measured with the following item: “I considered the training informative”. Directly after the training, supervisors were asked to rate this item on an evaluation form. Response categories ranged from 1 (totally disagree) to 5 (totally agree). The percentage of supervisors that found the training informative (agreed or totally agreed) was calculated. Supervisors could also report any particular comments about the PA in this evaluation form.

#### Fidelity

##### Organisational level

Fidelity was defined as the extent to which the working group meetings were performed according to the protocol. A researcher (RAK) was present during the working group meetings and registered whether (1) an in-company OHP chaired the working group meeting, (2) all four earlier described topics were discussed, and (3) all stakeholder representatives participated in the group discussions.

##### Supervisor level

Fidelity was defined as the extent to which the 4-h training was performed according to the protocol. At each training session, a researcher (RAK, FGS or CRLB) was present who registered whether (1) the training was delivered by in-company OHPs, (2) all four earlier described topics were discussed, and (3) all supervisors participated in the group discussions and role-playing.

#### Satisfaction

##### Organisational level

The stakeholder representatives’ appraisal of the working group meeting was used as parameter of satisfaction at the organisational level. Directly after the working group meeting, stakeholder representatives were asked to rate the working group meeting (scale 0–10) on an evaluation form.

##### Supervisor level

At the supervisor level, satisfaction was defined as the supervisors’ appraisal of the 4-h training. Directly after the training, supervisors were asked to rate the training (scale 0–10) on an evaluation form.

#### Barriers to application of the participatory approach

During the working group meetings, stakeholder representatives were asked about possible barriers to supervisors’ application of the PA within their organisation. If barriers were put forward, stakeholder representatives were also invited to think of possible solutions for these barriers.

#### Application of the participatory approach (PA)

At baseline, supervisors’ application of the PA was measured with two questions: “Are you familiar with the PA?” and “Have you applied the PA in the past 6 months?” At 6-month follow-up, supervisors were asked whether they knew when and how to apply the PA, how often they had applied the PA during the 6-month follow-up period, and reasons for not having applied the PA.

### Data collection and statistical analysis

Data for process variables were obtained via logs and evaluation forms of working group meetings and supervisor training sessions, online questionnaires at baseline and at 6-month follow-up, and interviews with HR professionals and OHPs. All data were analysed using descriptive statistics.

## Results

### Study participants

Nineteen stakeholder representatives and 49 supervisors participated in the study (more information is provided further below). The mean age of the representatives was 46 years (SD = 9) and 21 % were male. After the working group meetings, 18 stakeholder representatives (95 %) filled out an evaluation form. At baseline, 49 supervisors filled out the questionnaire, while 41 supervisors filled out the follow-up questionnaire after 6 months (loss to follow-up = 16 %). The mean age of supervisors (*n* = 49) was 47 years (SD = 7) and 61 % were male. More than 75 % of the participating supervisors had a high level of education. On average, the supervisors had 11 years (SD = 7) of supervisory experience and 29 subordinates (SD = 23). After the training, 47 supervisors (96 %) filled out an evaluation form.

### Context

Participating organisations differed in size: the steel factory employed about 9000 employees, the university medical centre about 5800 employees, and the university about 4600 employees. During the study, the steel factory and the university underwent reorganisation with downsizing of staff. Sick-leave rates (% of lost calendar days within 1 year) in 2012 were 4.6 % in the steel factory, 3.5 % in the university medical centre, and 4.7 % in the university. At the steel factory and the university medical centre, the RTW case management role was fulfilled by a supervisor, whereas at the university this role was fulfilled by an HR advisor. The supervisor training and coaching were delivered by labour experts at the steel factory, by company social workers at the university medical centre, and by occupational health nurses at the university.

### Recruitment

#### Organisational level

At the start of the study, the HR director of each organisation signed a letter of intent of participation. Within each organisation, a contact person was assigned for the implementation of the PA. Contact persons of the steel factory, the university medical centre, and the university were two labour experts (the trainers), a HR staff advisor, and the head of the occupational health service, respectively. In all organisations, stakeholder representatives were suggested by the contact person, and subsequently approached by one of the researchers (RAK) to participate in the working group.

#### Supervisor level

Within all three organisations, supervisors were initially not directly approached by the researchers. At the steel factory, the contact persons were assisted by HR advisors in making an inventory of supervisors who might be interested in the training. At the university medical centre and university, the department managers and HR advisors of all departments were informed about the study in personal meetings, in which they were asked to recruit supervisors for the study. Department managers and HR advisors provided a list with supervisors who might be interested in the training. Subsequently, eligible supervisors were informed about the study protocol by the research team via e-mail, and received an invitation for the supervisor training. If supervisors wanted to participate in the training, they first had to fill out the baseline questionnaire.

### Reach

#### Organisational level

In total, 19 out of 24 approached stakeholder representatives (79 %) attended the working group meetings (see Table 1). Due to organisational structures, some stakeholders were represented by more than one representative. At the steel factory, four out of six stakeholders were represented: no one represented the OPs and department managers. At both the university and the university medical centre, five out of six stakeholders were represented: there was no representative of the department managers. The main reason mentioned for non-participation was: “not available due to other appointments”.

**Table 1:** Results of process variables at organisational and supervisor level

	Total	Steel factory	University Medical Centre	University
*Organisational level*				
Reach (%)	19/24 (79 %)	5/7 (71 %)	8/9 (89 %)	6/8 (75 %)
Number of represented stakeholders (maximum is 6)		4 (no OPs or department managers)	5 (no department managers)	5 (no department managers)
	*n* = *18*	*n* = *5*	*n* = *8*	*n* = *5*
Dose delivered: number of working group meetings	3	1	1	1
Dose received: active participation (%)	72	100	62	60
Satisfaction (0–10), M (SD)	7.5 (1.0)	8.2 (0.4)	7.0 (1.0)	7.7 (1.2)
*Supervisor level*				
Reach (%)	116/1050 (11 %)	62/344 (18 %)	44/390 (11 %)	10/330 (3 %)
	*n* = *47*	*n* = *25*	*n* = *15*	*n* = *7*
Dose delivered: number of 4-h training sessions (T) and coaching sessions (C), *n*	T 9; C 3	T 4; C 0	T 3; C 2	T 2; C 1
Dose received: considered training informative (%)	98	96	100	100
Satisfaction regarding training (0–10), M (SD)	7.7 (0.6)	7.5 (0.6)	7.8 (0.6)	8.2 (0.8)

#### Supervisor level

In total, 116 out of 1050 approached supervisors (11 %) enrolled in the study. Sixty-one supervisors were allocated to the intervention group, of whom 49 supervisors attended the training and three supervisors were coached (see Table 1). Not all supervisors in the intervention group could attend the training because of other appointments. Main reasons mentioned for non-participation reported by department managers and/or HR advisors were: “sick leave at department level is not a priority at the moment”, “department is undergoing reorganisation”, and “supervisors do not need training or are too busy”.

### Dose delivered

In total, three 2-h working group meetings were delivered as intended: one per organisation. Each working group meeting was delivered approximately 1 month before the supervisor training. In total, nine 4-h training sessions, no follow-up training sessions, and three coaching sessions for supervisors were delivered (see Table 1).

### Dose received

In total, 13 out of 18 the stakeholder representatives (72 %) indicated that they had actively participated during the working group meeting.

In total, 46 out of 47 supervisors (98 %) indicated that they found the training informative (see Table 1).

### Fidelity

#### Organisational level

Most of the working group meetings were not chaired by only an in-company OHP: the working group meetings of the university medical centre and the university were (co-) chaired by a researcher (RAK). The in-company OHPs of the university medical centre and university indicated that they preferred a researcher as to chair the meeting because the researcher knew more about the application of the PA than they did. In all working group meetings, the four required topics were discussed and all stakeholder representatives who were present participated in the group discussions.

#### Supervisor level

All 4-h training sessions were given by in-company OHPs. In all training sessions, the four required topics were discussed and all supervisors who were present participated in the group discussions. However, at the steel factory and the university medical centre, respectively, 30 and 70 % of the training sessions included role-plays as specified in the protocol.

### Satisfaction

Stakeholder representatives rated the working group meeting with a mean score of 7.5 (SD = 1.0). Overall satisfaction was good; however, three representatives stated that in future working groups more supervisor representatives should be present as they are the main target group. Supervisors positively rated the training with a mean score of 7.7 (SD = 0.6) (see Table 1). Particularly, the role plays were positively appreciated, and it was suggested to use these even more in future trainings. One of the supervisors stated the following: “At first, I was not sure how to stick to the protocol, but with the role-plays it became clearer to me. And it turns out to be a nice working method”.

### Barriers to the application of the participatory approach (PA)

Stakeholder representatives of the university medical centre and university did not mention serious barriers to the application of the PA within their organisation. Stakeholder representatives of the steel factory reported “supervisors’ workload due to other supervisory tasks”, and “fewer work adjustments or solutions possible due to the reorganisation” as possible barriers to supervisors’ application of the PA within their organisation. However, during the working group meeting it was agreed that these barriers could not be solved at that time.

### Application of the participatory approach (PA)

At baseline, eight of the 49 participating supervisors (16 %) were already familiar with the PA, and four supervisors (8 %) had applied the PA once before. At 6-month follow-up, 38 supervisors (93 %) still knew when and how to apply the PA, and 11 supervisors (27 %) had applied the PA at least once during the 6-month follow-up period. In total, these 11 supervisors had applied the PA 22 times. Supervisors reported different reasons for not applying the PA: “employee’s sick leave is not sufficiently serious to apply the PA”, “the reason for sick leave is very clear such as a broken leg”, “the application of the PA is too time-consuming”, “the PA has not yet been incorporated in the departments’ policy”, and “the application of the PA was too difficult for employees”. This last reason was agreed upon by supervisors who did apply the PA with their employees as is illustrated with the following statement: “For my employee filling out the PA form was too difficult. However, the conversation did help him to put things in perspective”.

## Discussion

This study systematically evaluated the process of a multifaceted strategy to implement the PA within three organisations. Not all appointed stakeholders were represented in the working group meetings, and only 11 % of supervisors could be reached. The working group meetings and the supervisor training were delivered and received as planned and were well appreciated within all three organisations. Overall, the multifaceted implementation strategy was carried out as intended, except for the reach at organisational and supervisor level.

### Explanation of findings and comparison with other studies

The reach at supervisor level (11 %) was low compared to other studies on supervisor training that had a reach of 54–100 % (Coffeng et al. 2013; Takao et al. 2006; Theorell et al. 2001). Several explanations are possible for this low reach. First, participation was not mandatory for supervisors and therefore they may have given it less priority when in lack of time due to other tasks. Second, within the university the reorganisation clearly impeded the recruitment of supervisors, because the reorganisation was the main reason for department heads not to recruit supervisors at all. In addition, the reorganisation within the steel factory and university may also have caused fewer supervisors to enrol in the study because of extra work due to the reorganisation (Nielsen et al. 2010). Third, in the university an HR advisor is appointed as RTW case manager instead of the supervisor. Therefore, university supervisors may not feel responsible for the prevention and coordination of sick leave, which might explain the particularly low reach of 3 % within the university. Nevertheless, it should also be taken into account that we aimed to include 107 supervisors (10 %) in the randomised controlled trial (Kraaijeveld et al. 2013) and it was not attempted to increase this number.

The percentage of approached stakeholder representatives that attended a working group meeting was 79 %. This reach at organisational level is similar to that reported in other studies using a comparable working group component (Driessen et al. 2010; van der Meer et al. 2014; Pehkonen et al. 2009). It is remarkable that within all participating organisations no management representative was present at the working group meetings. The absence of a management representative could have affected the implementation of the PA, as commitment of the higher management has been recognised as an important driver for successful implementation (Murta et al. 2007; Coffeng et al. 2013; Egan et al. 2009). Unfortunately, the reason for their absence is unknown. To gain commitment, more attention should be paid to make them realise the importance of their involvement in the implementation strategy.

Hardly any supervisor coaching was delivered, as only three of the 49 supervisors were coached during the follow-up period. Although the coaching was available on request and supervisors were not required to make use of it, we had expected more supervisors to take advantage of the opportunity to receive personal coaching for the application of the PA. Other studies on employee health promotion have shown that in addition to supervisor training, supervisor coaching can offer a valuable contribution (Coffeng et al. 2014; Karlqvist and Gard 2013). As we do not know the reason for the limited delivery of supervisor coaching in this study, this requires more research.

The fidelity of the supervisor training is considered sufficient as all 4-h training sessions were almost completely performed according to the protocol: only the role-playing was not performed according to the protocol as this was not fully carried out in all training sessions. In the university and the university medical centre, almost all supervisors participated in the role-playing, while in the steel factory role-play was included in only 30 % of the training sessions. Role-playing can contribute to learning communication skills (Bosse et al. 2012), as it offers supervisors the possibility to practise and receive feedback from peers. In our training sessions, small groups of supervisors engaging in role-plays simultaneously did not always lead to active participation from all participants. In these cases, it was decided to perform plenary role-plays with several volunteering supervisors. This meant that in these training sessions, not all supervisors engaged in role-playing. However, watching peers engage in role-playing might not increase self-efficacy regarding the new behaviour to the same extent as actively engaging in the role-playing.

At 6-month follow-up, the proportion of supervisors that actually had applied the PA within the last 6 months was only 27 %. Although participating supervisors were probably all confronted during this period with an employee at risk of sick leave or sick-listed, it seems that they do not always find the PA suitable for application to prevent or shorten sick leave of employees. Only one half-day training might have been insufficient to teach supervisors how to identify that an employee is it at risk of sick-leave and how to discuss this with the employee concerned. In addition, applying the PA also expects some degree of competence from the employee in analysing specific work functioning problems instead of thinking in terms of feeling unwell. Yet employees did not receive training within the multifaceted implementation study, which might have impeded applying the PA in all relevant situations.

### Strengths and limitations

This study has some important strengths. By using the framework of Linnan and Steckler (2002) the implementation process of the multifaceted strategy was systematically evaluated. The assessed process variables offer insight into the implementation process of the multifaceted strategy of the PA, and into the added value of the three separate components of the strategy to implement the PA. Furthermore, this study differentiated between organisational and supervisor level, which resulted in a better understanding of the multifaceted implementation strategy at both levels within three different organisations. Lastly, the process data are examined separately from the results of the effectiveness study on the multifaceted implementation strategy of the PA, and the process data can therefore be interpreted without any bias of the results of the effectiveness study (Murta et al. 2007).

Some limitations should also be considered. First, due to the recruitment strategy of supervisors via department managers and HR advisors, we are unsure whether all 1050 eligible supervisors were indeed invited for participation. We believe that department managers decided whether their department would participate and that they also appointed specific supervisors to participate. This complicated calculating the percentage of our reach of supervisors. Second, for the fidelity of the working group meetings and supervisor training a researcher only observed whether both were performed according to the protocol. Fidelity could have been measured more extensively in order to be able to calculate a total score and a percentage for fidelity, for instance by using a checklist with scorings for the topics, the group discussions, and the role-playing. This might have made differences in fidelity between the organisations more clear.

### Implications for research and practice

The study reveals several implications for research and practice. First of all, it shows that it is important to select the right time for implementation of a new approach, as within the steel factory and the university a reorganisation probably impeded the implementation of the PA (Curran et al. 2012). A study of Nielsen and colleagues (2010) showed that ongoing changes within an organisation made it difficult for supervisors to focus on intervention implementation. Future studies should therefore carefully consider the organisational context and the readiness for implementation within an organisation. Second, it should be considered to train supervisors more extensively, but also to offer employees support or training in their role within the PA, when their supervisor suggests to apply the PA to tackle their work functioning problems. A study by Linden et al. (2014) showed that an occupational health care management program focusing on all employees within a department was successful in increasing self-efficacy of these employees to discuss necessary work changes to prevent work stress or absenteeism. Besides the supervisor, an important expert in the prevention of sick leave is the occupational physician. He or she may help the supervisor by giving the necessary medical information required for an adequate case management. The role of the occupational physician in the participatory approach to prevent sick leave should be explored in future research.

Furthermore, the supervisor coaching component should be reconsidered. It should be investigated why very few supervisors made use of the optional personal coaching, whether they experience a need for coaching and if so, how this could be delivered better. Last, even if an implementation strategy is mainly focused on a specific level of the organisation, it is nevertheless recommended to carefully consider measurement of process data on all different levels within an organisation in future process evaluations of implementation strategies, including the level of employees.

## Conclusion

The process evaluation showed that the multifaceted implementation strategy was predominantly carried out as intended. Reach at organisational level was reasonable, but no department manager was represented in the working group meetings. Reach at supervisor level was low. The working group meetings and supervisor training were delivered within all three organisations as planned; however, hardly any supervisor coaching was delivered. To further increase application of the PA by supervisors to prevent or shorten sick leave, it is recommended to engage department managers more actively and to enhance the training of both supervisors and employees.
